# (1→3)-β-D-glucanemia in deep mycosis

**DOI:** 10.1080/09629359791604

**Published:** 1997-11

**Authors:** Taminori Obayashi

**Affiliations:** 1 Department of Clinical Pathology Jichi Medical School 3311-1 Yakushiji Minamikawachi-machi, Kawachi-gun Tochigi 329-04 Japan

## Abstract

Factor G, a coagulation proenzyme of the Japanese horseshoe crab (*Tachypleus tridentatus*), is extremely sensitive to (1→3)-β-D-glucan, which is a characteristic cell-wall constituent of fungi. Using this factor and by a digestion study with (1→3)-β-D-glucan, we showed that blood from patients with deep m ycosis contains the glucan. It was detected in 39 out of 41 fungal febrile episodes (sensitivity 90%), but in none of the 59 nonfungal febrile episodes (specificity 100%).


Mediators of Inflammation, 6, 271–273 (1997)

### (1→3)-β-D-glucanemia in deep mycosis

**Taminori Obayashi**

Department of Clinical Pathology, Jichi Medical School, 3311-1 Yakushiji, Minamikawachi-machi, Kawachi-gun, Tochigi 329-04, Japan

Tel: +81 285 44 2111
Fax: +81 285 44 9947

**Factor G, a coagulation proenzyme of the Japanese horseshoe crab (*Tachypleus tridentatus*), is extremely sensitive to (1→3)-β-D-glucan, which is a characteristic cell-wall constituent of fungi. Using this factor and by a digestion study with (1→3)-β-D-glucan, we showed that blood from patients with deep mycosis contains the glucan. It was detected in 39 out of 41 fungal febrile episodes (sensitivity 90%), but in none of the 59 non-fungal febrile episodes (specificity 100%).**

**Keywords:** Fungal infections, endotoxin

### Introduction

(1→3)-β-D-glucan-like chitin is a characteristic cell-wall component of almost all fungi [1]. Other micro-organisms such as bacteria, rikettsiae and viruses all lack this polysaccharide, as do the cells and extracellular fluids of animals. This makes it a good indicator of systemic fungal infections, if detected in the blood of animals.

A method that can determine the concentration of (1→3)-β-D-glucan with high sensitivity and specificity has recently become available. The test uses the special coagulation factor of the Japanese horseshoe crab *(Tachypleus tridentatus)* which is extremely sensitive to the polysaccharide. This paper outlines results obtained by applying the test to the diagnosis of deep mycosis.

### Materials and methods

(1→3)-β-D-glucan was determined with the Fungitec G test (Seikagaku Corporation, Tokyo, Japan) according to the manufacturer's instructions. The standard (1→3)-β-D-glucan was that derived from *Poria cocos*.

### Reactivity of the G test to polysaccharides extracted from different fungi

Cells of *Candida albicans*, *Microsporum canis*, *Trichophyton mentagrophytes*, *T. rubrum*, *Saccharomyces cerevisiae*, *Aspergillus flavus*, *A. fumigatus* and *Cryptococcus neoformans* were autoclaved at 121°C for 90 min. The hot-water extracts were then centrifuged, and the supernatant was subjected to the G test. The sugar content of the supernatants was determined with the phenol-sulfate method [2].

### Plasma (1→3)-β-D-glucan assay

(1→3)-β-D-glucan concentrations of plasmas obtained from patients with fungemia were determined and compared with those of patients with endotoxemia and normal individuals. Samples were pretreated with perchloric acid as described elsewhere [3].

### Digestion of G test-reactive plasmas with (1→3)-β-D-glucanase

Plasmas that showed a positive reaction were digested with (1→3)-β-D-glucan purified from Zymolyase (Seikagaku Corporation, Tokyo, Japan) to confirm that the reactive substance in those samples was (1→3)-β-D-glucan.

### Sensitivity and specificity of the G test for the diagnosis of deep mycosis

Plasma (1→3)-β-D-glucan concentrations were determined in blood cultures taken during 202 febrile episodes in 179 patients. The standard requirement for a diagnosis of deep mycosis was an autopsy or a microbiological examination of appropriate specimens such as blood and cerebrospinal fluid.

### Results

### Reactivity of the G test to fungal polysaccharide extracts

The G test reacted in a dose-dependent manner with every fungal extract, although there was a difference in reactivity between the species (Fig. 1).

© 1997 Rapid Science Publishers

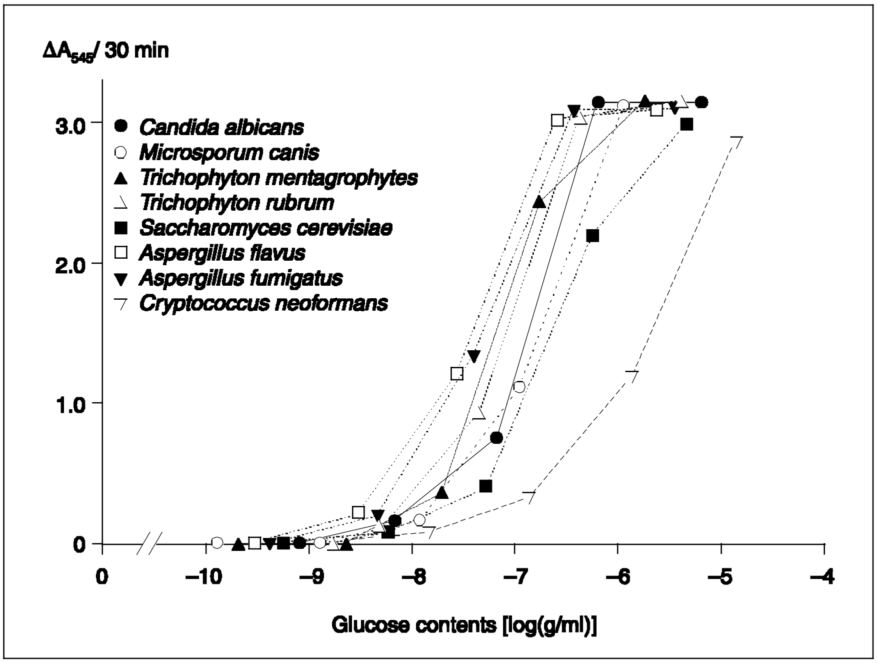

FIG. 1. Reactivity of the G test to various fungal polysaccharides.

**Table 1.** Comparison of plasma (1→3)-β-D-glucan concentrations (pg/ml) between fungemia and endotoxemia

| Fungemia | | Endotoxemia | |
|---|---|---|---|
| 1 | 269 | 1 | 1.4 |
| 2 | 1733 | 2 | 0.6 |
| 3 | 329 | 3 | 1.2 |
| 4 | 1719 | 4 | 0.2 |
| 5 | 167 | 5 | 0.8 |
| | | 6 | 0.9 |
| | | 7 | 0.2 |

Normal ($n$ = 20): 0.2 ± 0.3 pg/ml.

**Table 2.** G-test reactivity of plasma from patients with deep mycosis before and after digestion with endo-(1→3)-β-D-glucanase

| Case no. | Before digestion $\Delta A_{545}$/30 min | After digestion $\Delta A_{545}$/30 min | Residual activity (%) |
|---|---|---|---|
| 1 | 1.244 | 0.016 | 1.3 |
| 2 | 1.627 | 0.008 | 0.5 |
| 3 | 2.185 | 0.013 | 0.6 |
| 4 | 1.245 | 0.000 | 0.0 |
| 5 | 1.215 | 0.001 | 0.5 |
| Normal | 0.005 | 0.001 | – |

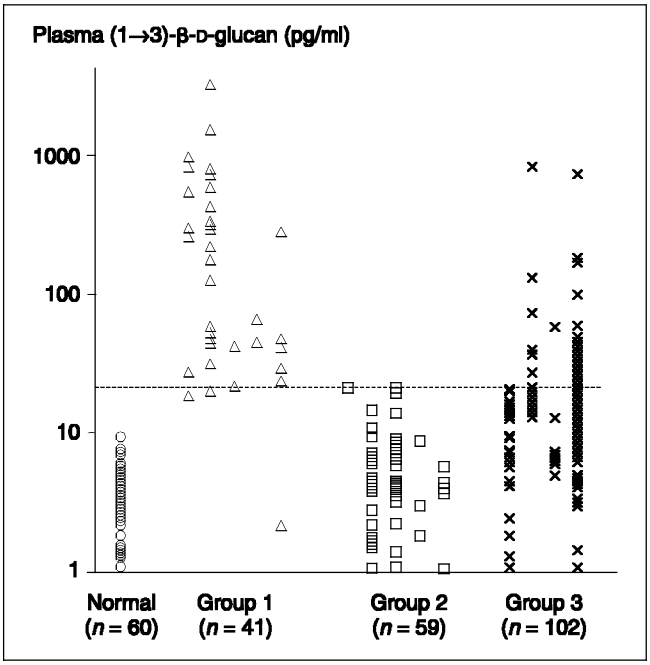

FIG. 2. Plasma (1→3)-β-D-glucan concentrations in febrile hospitalized patients. Group 1, other fungal infections (6), fungal meningitis (2), fungal cath fever (2), fungemia (22), fungemia + bacteremia (2), invasive deep mycosis (7); group 2, tumor fever (4), collagen disease (3), non-fungal non-bacterial infection (3), other bacterial infection (26), bacteremia (21), bacterial cath fever (1), systemic bacterial infection (1); group 3, fever of unknown etiology (59), culture-negative catheter fever (5), antifungal treatment effective (12), antibacterial treatment effective (26).

### Plasma (1→3)-β-D-glucan concentrations in fungal infections

(1→3)-β-D-glucan concentrations were elevated in fungal infections, while they were normal in endotoxemia and in healthy controls (Table 1).

### Digestion study with (1→3)-β-D-glucanase

The high absorbance of plasmas from patients with fungal infections became almost null after digestion with (1→3)-β-D-glucanase (Table 2).

### Sensitivity and specificity of the G test

Of 202 febrile episodes, 41 were present in patients with deep fungal infections, 59 were of non-fungal etiologies and 102 were unknown. With a cut-off value at 20 pg/ml, 37 of the 41 fungal infections were positive, and none of the 59 non-fungal febrile episodes were positive. The sensitivity was thus 90% and the specificity 100% (Fig. 2).

### Discussion

Horseshoe crabs appear to defend themselves from fungal invasion with a specially designed protein stored in their hemocytes. Although the principal function of this protein (factor G) lies in hemostasis, the defense against fungal invasion is activated when (1→3)-β-D-glucan is released from fungi and then blood coagulation is initiated to prevent the spread of germs into the circulation. Factor G has recently been applied to the detection of (1→3)-β-D-glucan in biological fluids, and consequently to the diagnosis of deep mycosis.

The G test reacted directly with sugar extracts from different fungi. It yielded positive results with blood specimens from patients deeply infected with fungi, but not with those from patients with bacteremia. The disappearance of the reactivity from the blood after treatment with (1→3)-β-D-glucanase confirmed that (1→3)-β-D-glucan was present in the blood samples [4].

Because these preliminary results all indicated that the test was valid as a diagnostic aid in deep mycosis, we examined its sensitivity and specificity against febrile episodes in hospitalized patients [5]. Although normal concentrations of plasma (1→3)-β-D-glucan never exceeded 10 pg/ml, fungal febrile episodes appeared to be most clearly separated from non-fungal episodes by a cut-off line at 20 pg/ml. With this cut-off value, the sensitivity was 90% and the specificity 100%. This high specificity will help prevent indiscriminate use of antifungal agents and avoid the development of resistant strains. This is very important today, as there are only a few antifungals at hand for treatment. Unfortunately, the positive and negative predictive values were difficult to determine, because no standards are available that can clearly differentiate fungal from non-fungal infections. Development of such a standard and further accumulation of the data will establish the validity of the test.

### References

1. Reiss E: *Molecular Immunology of Mycotic and Actinomycotic Infections*. New York: Elsevier; 1986.
2. Dubois M, Gilles KA, Hamilton JK, Rebers PA, Smith F: Colorimetric method for determination of sugars and related substances. *Anal Chem* 1956, **28**:350–356.
3. Obayashi T, Tamura H, Tanaka S, Ohki M, Takahashi S, Kawai T: Endotoxin activating activity in normal and pathological human blood samples. *Infect Immun* 1986, **53**:294–297.
4. Obayashi T, Yoshida M, Tamura H, Aketagawa J, Tanaka S, Kawai T: Determination of plasma (1→3)-β-D-glucan: a new diagnostic aid to deep mycosis. *J Med Vet Mycol* 1992, **30**:275–280.
5. Obayashi T, Yoshida M, Mori T, *et al.*: Plasma (1→3)-β-D-glucan measurement in diagnosis of invasive deep mycosis and fungal febrile episodes. *Lancet* 1995, **345**:17–20.
